# Correcting gut dysbiosis can ameliorate inflammation and promote remyelination in multiple sclerosis – Commentary

**DOI:** 10.1177/13524585211018990

**Published:** 2021-05-28

**Authors:** R Hohlfeld

**Affiliations:** Institut für klinische Neuroimmunologie, LMU Klinikum, Ludwig-Maximilians-Universität München, München, Germany/Munich Cluster for Systems Neurology (SyNergy), Munich, Germany

Basic research has uncovered surprising connections between the gut microbiota and essential functions of the body. These exciting findings offer fascinating perspectives for the treatment of human diseases. Clinical application, however, lags behind the progress made in basic understanding of the microbiota. This sobering fact provides the background for the current controversy.

Laura Calvo-Barreiro and colleagues argue that therapeutic modulation of the microbiota can not only regulate peripheral immune responses but also induce remyelination and neuroprotection.^1^ This optimistic view is supported by proponents’ studies in animal models.^2,3^ By contrast, Christopher McMurran points out that remedying dysbiosis can improve inflammation but not demyelination in animal models.^4^ His more sceptical view is based on his observation that modulation of the microbiota had only minimal impact on remyelination in toxic-induced (lysolecithin or cuprizone) mouse models.^5^

Taken together, there is convincing evidence that modulation of the microbiota can ameliorate (experimental) autoimmune responses. There is also firm evidence that the microbiota regulates neurobiological and microglial functions via the ‘gut–brain axis’.^6^ It is less clear, however, whether the microbiota has a direct influence on myelination.

What are the most urgent challenges for *human* microbiota research to push the field towards clinical translation? First, the number of studied subjects needs to be drastically increased. Many published studies indicate that in multiple sclerosis (MS) there is indeed a ‘dysbiosis’ of the commensal microbiota.^7^ However, most of these studies were based on relatively small cohorts lacking rigorous controls. In this regard, the international MS Microbiome Study (iMSMS) takes a big step forward by aiming to collect a very large number of samples from people with MS. Household members serve as controls.^8^ This large study should help to characterize the currently somewhat vaguely described state of ‘dysbiosis’. Second, in order to identify disease-relevant microbes, it is essential that candidate bacteria are tested for their *functional* disease-promoting capacity. This can be achieved, for example, with gnotobiotic mouse models: colonization of genetically engineered autoimmune-prone, germ-free mice with human-derived microbiota helps to distinguish between disease-promoting and protective bacteria.^9,10^ Identification of an MS-relevant microbial signature should open new doors for rational therapy.
